# Covid‐19 Spike protein impact on Alzheimer's (AD) and Cerebrovascular Disease

**DOI:** 10.1002/alz70855_106266

**Published:** 2025-12-24

**Authors:** Sally A. Frautschy, Kapil Manglani, Xiaohong Zuo, Cansheng Zhu, Alex Bombino, Johnathan Kwon, Mychica Jones, Douglas Fraser, Gregory M Cole

**Affiliations:** ^1^ Veterans Greater Los Angeles Healthcare System, Los Angeles, CA, USA; ^2^ University of California Los Angeles, Los Angeles, CA, USA; ^3^ Veteran Administration Geriatric Education Clinical Center, Los Angeles, CA, USA; ^4^ University of California, Los Angeles, Los Angeles, CA, USA; ^5^ Veterans Administration Geriatric Education and Clinical Center, Los Angeles, CA, USA; ^6^ Veterans Administration, Geriatric Research and Education Center, Los Angeles, CA, USA; ^7^ Western University, London, ON, Canada

## Abstract

**Background:**

Most COVID‐19 cases fully recover, but 7% report persistent symptoms (Long Covid, LC). SARS‐CoV2 uses surface S1 Spike protein to bind ACE2 receptors leading to sustained complement activation. S1 activates endothelial C3, leukocyte attachment and invasion, also observed in atherosclerosis, cerebral amyloid angiopathy (CAA), and hypertension‐driven small vessel disease (CSVD). LC patients have elevated plasma AD biomarkers and postmortem evidence of microglial, complement and microvascular pathology. AD patients are at increased risk for Neuro‐Covid particularly those with ApoE4 and hypertension. Here we explore modulatory roles of hypertension or ApoE4 on the response to S1 in AD rats.

**Method:**

We created rat models with AD or mixed AD (AD with CSVD) with or without ApoE4. Outcomes included executive function, AD pathology, white matter damage and complement activation 6 weeks after subcutaneous injection of recombinant S1. We developed high throughput plasma assays to assess vascular and CNS complement activation from rat models or human Neuro‐Covid.

**Result:**

Experiment 1: Independent of human ApoE or hypertension, S1 increased plaque and vascular amyloid and amyloid responsive ptau217, microvascular active C3 (C3d) and neuronal and vascular complement C5b‐9 terminal attack complex. E4 and hypertension synergized to increase vascular pathology induced by spike. This was accompanied by leukocyte adhesion, including neutrophils. Experiment 2: S1 spike injected 11‐13 month rats showed spike‐induced executive function deficits and significant increases in the hypertensive FAD rats for beta amyloidogenic APP BACE processing, ptau217 as well as loss of myelin basic protein, an index of white matter damage. We also observed increased cortical active C3, CSF C5a (ELISA), and active C3 in plasma endothelial and astrocyte‐derived EV. Pre‐existing hypertensive comorbidity potentiated Covid spike effects. The plasma of LC patients with Neuro‐Covid showed elevated active C3 in astrocyte and endothelial EV.

**Conclusion:**

Spike protein caused robust increases in AD pathology as well as persistent pathogenic CNS and vascular complement activation, associated with synapse loss and BBB damage in rats with pre‐existing AD and vascular pathology, a situation found in at least 20 to 30% of our aged population. Scalable assays for Neuro‐Covid complement activation should facilitate treatment.

